# What motivates individuals to share information with governments when adopting health technologies during the COVID-19 pandemic?

**DOI:** 10.1186/s12889-023-17437-2

**Published:** 2023-12-18

**Authors:** Zixuan Peng, Prossy Kiddu Namyalo, Xu Chen, Mingjie Lv, Peter C. Coyte

**Affiliations:** 1 School of Public Health, Southeast University, Nanjing, China; 2 Institute of Health Policy, Management and Evaluation, University of Toronto, Toronto, Canada; 3 Zhejiang Provincial Philosophy and Social Sciences Pilot Laboratory, Hangzhou, China; 4 Laboratory of Intelligent Society and Governance, Zhejiang Lab, Hangzhou, China

**Keywords:** COVID-19, Digital governance, Information sharing intentions

## Abstract

**Background:**

While digital governance has been adopted by governments around the world to assist in the management of the COVID-19 pandemic, the effectiveness of its implementation relies on the collection and use of personal information. This study examines the willingness of individuals to engage in information-sharing with governments when adopting health technologies during the COVID-19 pandemic.

**Methods:**

Data were obtained from a cross-sectional survey of 4,800 individuals drawn from 16 cities in China in 2021. Tobit regression models were used to assess the impacts of an array of determinants on an individual’s willingness to share information with governments when adopting health technologies.

**Results:**

Individuals who perceived a higher level of helpfulness, risk, expectations from others, weariness toward privacy issues, and were sensitive to positive outcomes were more willing to share information with governments when adopting health technologies during the COVID-19 pandemic. Across all the subgroups, self-efficacy only reduced the willingness to share information with governments for individuals who spent more than seven hours per day online. The negative impacts of being sensitive to negative outcomes on the willingness to share information were only found among females and the less educated group.

**Conclusions:**

This study revealed the seemingly paradoxical behavior of individuals who perceived high risks of sharing information and a sense of fatigue toward privacy issues yet continued to be willing to share their information with their governments when adopting health technologies during the COVID-19 pandemic. This work highlighted significant differential motivations for sharing information with governments when using health technologies during a pandemic. Tailored policies that resonate with population sub-groups were suggested to be proposed to facilitate crisis management in future situations.

**Supplementary Information:**

The online version contains supplementary material available at 10.1186/s12889-023-17437-2.

## Background

The COVID-19 pandemic has ravaged the world for the past four years [1]. This pandemic has been associated with a range of non-pharmacological responses that have had significant impacts on the physical and mental health of mankind [2–4]. Digital governance, defined as the use of information and communication technologies to improve organizational performance [5], has been employed by many governments to combat the pandemic [6, 7]. Digital governance was implemented by leveraging a wide range of specific interventions, such as the introduction and use of telemedicine, digital learning packages, and tracking and home monitoring technologies [7–10]. The importance of applying digital governance to enable individuals to make informed choices in the context of the pandemic has been stressed [8, 9, 11], but its implementation can be impeded by the unwillingness of individuals to share their information with governments when adopting health technologies [12, 13].

In the context of the COVID-19 pandemic, some studies have investigated factors impacting individuals’ willingness to travel [14], individuals’ willingness to share misinformation on social media [15–19], individuals’ willingness to share individual participant-level data from COVID-19 interventional trials [20], and restaurant customers’ willingness to disclose personal information [21]. However, there has been a paucity of research dealing with individuals’ information-sharing with governments when adopting health technologies during the COVID-19 pandemic. China implemented a set of strict COVID-19 containment policies [22, 23] and made extensive use of digital health tools to manage the pandemic. For example, the Chinese government introduced the Health Code, an e-passport that documents rapid antigen test results, medical records, and travel history [24–26], and many provincial health authorities worked with telecommunications and digital health enterprises to offer virtual care [26]. Within this context, it is useful to investigate the determinants of the willingness of citizens to share their personal information with governments when adopting health technologies during the COVID-19 pandemic.

Taken together, this study aims to explore and estimate the determinants of the willingness to share information with governments when adopting health technologies during the COVID-19 pandemic in China. The rest of the manuscript is structured as follows: In the next section, we introduced the conceptual model that will be used to guide our empirical analyses. In Sect. 3, we describe the data and statistical methods employed. The key findings are presented in Sect. 4 before being discussed in Sect. 5. We end with a brief set of conclusions and offer a set of policy suggestions to aid future pandemic crisis situations.

## Conceptual model

This study draws on the theory of reasoned action (TRA) [27], the theory of Planned Behavior (TPB) [28], the privacy calculus theory (PCT) [29, 30], the regulatory focus theory (RFT) [31] as well as theories and literature [32, 33] that focus on exploring determinants of individuals’ information-sharing intentions to frame our conceptual model.

TRA was developed by Fishbein and Ajzen (1975) [27] and was extended by Ajzen (1991) to become TPB [28]. TRA and TPB focus on factors, including attitudes, subjective norms, and perceived control, that impact the willingness of individuals to perform specific behaviors [34]. Privacy fatigue, as a measure of individuals’ attitudes [35], often results in an inability to make decisions regarding privacy issues so that individuals with privacy fatigue always want to minimize efforts in decision making [36]. Subjective norm refers to perceived expectations from others (such as family members, friends, colleagues, and other important persons) who have impacts on whether individuals will perform a particular behavior [37]. Self-efficacy refers to beliefs in an individual’s capabilities to mobilize motivation, cognitive resources, and courses of action required to achieve given goals [38]. Plenty of prior studies have demonstrated the positive impacts of privacy fatigue, subjective norms, and self-efficacy on individuals’ willingness to share information [35–39]. Based on this evidence, the following hypotheses were proposed:

### H 1

*Privacy fatigue positively affects an individual’s willingness to share information when adopting health technologies during the pandemic*.

### H 2

*Subjective norm positively affects an individual’s willingness to share information when adopting health technologies during the pandemic*.

### H 3

*Self-efficacy positively affects an individual’s willingness to share information when adopting health technologies during the pandemic*.

PCT was originally developed by Laufer and Wolfe (1977) [29] before being applied in the field of information practices by Culnan and Armstrong (1999) [30]. PCT suggests that individuals make privacy-based decisions by evaluating the benefits (perceived helpfulness) any information may bring against the risk (perceived risk) of its disclosure [40]. Plenty of prior studies have shown that individuals’ willingness to disclose information was negatively associated with the level of the perceived risk while being positively associated with the level of the perceived helpfulness [39, 41]. Based on this evidence, we advanced two hypotheses:

### H 4

*Perceived risk negatively affects an individual’s willingness to share information when adopting health technologies during the pandemic*.

### H 5

*Perceived helpfulness positively affects an individual’s willingness to share information when adopting health technologies during the pandemic*.

RFT was developed by Higgins [31] and focuses on how individuals achieve goals through self-regulation. RFT suggests that individuals can pursue goals with either a promotion or a prevention focus. Individuals are responsive to positive outcomes and opportunities for approaching goals when they are promotion-focused, while they are more responsive to negative consequences and goal impediments if they are prevention focused [42]. Promotion (prevention) focus has been shown to be positively (negatively) associated with the willingness to share information as individuals with a promotion (prevention) focus counted more on the positive (negative) outcomes of sharing information [43]. Prior studies have also demonstrated that individuals’ regulatory focus moderated the impacts of perceived helpfulness, perceived risk, and privacy fatigue on individuals’ willingness to share information [44]. Based on the above rationale and the well-established RFT model, we proposed the following hypotheses:

### H 6

*Promotion focus positively affects an individual’s willingness to share information when adopting health technologies during the pandemic*.

### H 7

*Prevention focus negatively affects an individual’s willingness to share information when adopting health technologies during the pandemic*.

### H 8

*Promotion and prevention focus moderate the impacts of perceived helpfulness, perceived risk, and fatigue on an individual’s willingness to share information when adopting health technologies during the pandemic*.

## Methods

### Data collection

The study adopted a survey design in which data were collected by researcher administered questionnaires distributed to people above 18 years and above. All the surveyors received professional training to ensure a correct understanding of the concepts and the content of the survey before administering the questionnaires in person and conducting face-to-face interviews in December 2021 across 16 cities in China 1. Our study samples included individuals aged more than 18 years and we adopted a quota sampling method to ensure that our research sample obtained from these quotas has similar proportions of observations in demographic characteristics as the whole population in China. Power calculations were performed to ensure sufficient study respondents to make population inferences. Although a sample size of 598 would ensure the study was powered to estimate the proportion of individuals willing to share their information, we increased the sample to 4,800 since regression methods were to be used. After removing invalid samples wherein the research participant answered more than 70% of the questions similarly and samples with missing values, we finally obtained an analysis sample comprising 4,800 individuals from the 16 cities in China 2.

### Measures of variables

The willingness of individuals to share information with governments when adopting health technologies was the outcome variable of interest. It was measured by answers to the question “Are you willing to share information with the governments when using health technologies during the pandemic?”, and a seven-point Likert scale ranging from strongly disagree to strongly agree was used to record responses. Perceived risk and helpfulness, subjective norm, privacy fatigue, self-efficacy, promotion and prevention focus were included as the key independent variables. These variables were measured by a set of previously validated scales (details of which are outlined in Table A.1 in Appendix A). All items used in these measurement scales were again rated on a seven-point Likert scale from “strongly disagree” to “strongly agree”. The sum score of the items used to measure perceived helpfulness, perceived risk, subjective norm, privacy fatigue, self-efficacy, promotion focus, and prevention focus ranged from 0 to 28, 0–21, 0–28, 0–28, 0–18, 0–42, and 0–28, respectively. A higher sum score denotes a higher level of perceived helpfulness, perceived risk, subjective norm, privacy fatigue, self-efficacy, promotion focus, and prevention focus. The Cronbach’s alpha coefficient of perceived helpfulness, perceived risk, subjective norm, privacy fatigue, and self-efficacy were 0.76, 0.84, 0.94, 0.86, and 0.80, respectively, implying a high degree of internal consistency of the items included in the measurement scales [45]. The Cronbach’s alpha coefficient of promotion and prevention focus was lower at 0.44 and 0.63, indicating that the items included are weakly related [45].

We also controlled for the impacts of a set of variables that have been shown to impact the willingness of individuals to share information [15, 16, 18, 22, 25, 26, 44], including age (18–29, 30–39, 40–49, 50–59, or ≥ 60), sex (male or female), education (primary school and below, high school, bachelor’s degree, or master’s degree and above), income (≤ 4,999 CNY, 5,000–9,999 CNY, 10,000–14,999 CNY, or ≥ 15,000 CNY), employment status (employed or unemployed), and daily digital content consumption (measured by whether the individual spent more than seven hours per day online).

### Data analysis

A set of statistical analyses were conducted to examine the impacts of the proposed factors on individuals’ willingness to share information when adopting health technologies. First, we performed a descriptive analysis to report the characteristics of the sampled individuals. We compared variations in the willingness to share information across different population groups (stratified by age, gender, education, income, employment status, and daily digital content consumption) using the Chi-squared test. Second, we performed multivariate regression techniques to estimate the impacts of perceived helpfulness, perceived risk, subjective norm, privacy fatigue, self-efficacy, prevention focus, and promotion focus on the willingness to share information. We examined the moderating effects of promotion and prevention focus on the impacts of perceived helpfulness, perceived risk, and privacy fatigue on the willingness to share information by including interaction terms in the regression models. Since the willingness of individuals to share information was measured by positive integers, a Tobit regression model (model 2) was constructed to correct for potential bias emanating from conventional regression methods (model 1). Finally, we conducted a series of sensitivity analyses to verify the robustness of our key research findings. To examine whether our research findings were sensitive to the degree of economic development across cities, we classified our study sample into four sub-samples according to the gross domestic product of each city. Subgroup analysis was also performed to assess heterogeneity stemming from the demographic characteristics of our sampled individuals. We finally constructed a Poisson regression model to offer an alternative estimation strategy to the Tobit regression model. All the statistical analyses were conducted using the R statistical language [46].

## Results

### Results of descriptive analysis

Table 1 reports the results of the descriptive analysis. The mean score for the willingness to share information was 4.65 with a SD of 1.25, which suggests that the sampled individuals somewhat agreed to share their personal information with the government. Most of our sampled individuals were aged between 30 and 39 years (23.00%), had a monthly income of less than 4,999 CNY (44.69%), had a bachelor’s degree (61.98%), were employed (98.77%), and spent less than seven hours per day online (84.94%). Table 2 reports variations in the willingness to share information by demographic characteristics. We did not detect any statistically significant difference in the willingness to share information across different age, gender, income, and education groups. In contrast, we found that individuals who were unemployed and spent more than seven hours per day online were less likely to share information during the COVID-19 pandemic than their counterparts.

**Table 1:** The results of descriptive analysis

*Nob: 4,800*	*Mean*	*SD*	*Min*	*Max*
***Dependent variable***				
Willingness to share information	4.649	1.246	0	6
***Independent variables***				
Perceived helpfulness	15.575	4.295	0	24
Perceived risk	12.091	3.718	0	18
Subjective norm	17.740	4.683	0	24
Fatigue	11.229	5.908	0	24
Self-efficacy	11.686	3.582	0	18
***Moderators***				
Promotion focus	21.354	4.797	0	36
Prevention focus	13.013	3.736	1	24
***Control variables***				
Age	≤ 29 years old		1,056	22.00%
	30–39 years old		1,104	23.00%
	40–49 years old		1,056	22.00%
	50–59 years old		912	19.00%
	≥ 60 years old		672	14.00%
Gender	Female		2,400	50.00%
	Male		2,400	50.00%
Income	≤ 4,999 CNY		2,145	44.69%
	5,000–9,999 CNY		1,732	36.08%
	10,000–14,999 CNY		563	11.73%
	≥ 15,000 CNY		360	7.50%
Education	Primary school and below		356	7.42%
	High school		1,308	27.25%
	Bachelor’s degree		2,975	61.98%
	Master’s degree and above		161	3.35%
Employment	Employed		4,741	98.77%
	Unemployed		59	1.23%
Digital content consumption	No: Spent less than seven hours per day online		4,077	84.94%
	Yes: Spent more than seven hours per day online		723	15.06%

**Note:** Nob represents number of observations.

**Table 2:** Variations in the willingness to share information by group

		*WTS = 0,1,2*		*WTS = 4*		*WTS = 5,6,7*		*P test*
		*N = 472*		*N = 291*		*N = 4,037*		
Age (%)	18–29 years	124	26.3	71	24.4	861	21.3	0.142
	30–39 years	106	22.5	55	18.9	943	23.4	
	40–49 years	98	20.8	58	19.9	900	22.3	
	50–59 years	88	18.6	64	22	760	18.8	
	≥ 60 years	56	11.9	43	14.8	573	14.2	
Gender (%)	Female	235	49.8	132	45.4	2,033	50.4	0.256
	Male	237	50.2	159	54.6	2,004	49.6	
Employment (%)	Employed	469	99.4	277	95.2	3,995	99	< 0.001
	Unemployed	3	0.6	14	4.8	42	1	
Income (%)	≤ 4,999 CNY	229	48.5	145	49.8	1,771	43.9	0.183
	5,000–9,999 CNY	161	34.1	101	34.7	1,470	36.4	
	10,000–14,999 CNY	48	10.2	30	10.3	485	12	
	≥ 15,000 CNY	34	7.2	15	5.2	311	7.7	
Education (%)	Primary school and below	44	9.3	17	5.8	295	7.3	0.241
	High school	135	28.6	86	29.6	1,087	26.9	
	Bachelor’s degree	274	58.1	175	60.1	2,526	62.6	
	Master’s degree and above	19	4	13	4.5	129	3.2	
Digital content consumption (%)	No	396	83.9	230	79	3,451	85.5	0.010
	Yes	76	16.1	61	21	586	14.5	

Note: WTS represents individuals’ willingness to share information

### Results of main analysis

Table 3 reports the impacts of various factors on the willingness to share information. We found that perceived helpfulness, perceived risk, subjective norm, privacy fatigue, self-efficacy, and promotion focus enhanced the willingness to share information. Specifically, for a unit increase in the score for privacy fatigue, subjective norm, self-efficacy, perceived helpfulness, perceived risk, promotion focus, an individual’s willingness to share information increased by 0.03 (95% CI: 0.004 and 0.06), 0.01 (95% CI: 0.004 and 0.02), 0.01 (95% CI: 0.00004 and 0.02), 0.13 (95% CI: 0.09 and 0.17), 0.10 (95% CI: 0.05 and 0.13), and 0.15 (95% CI: 0.11 and 0.18), respectively. In contrast, prevention focus reduced the willingness to share information, though such an association was not statistically significant.

**Table 3:** Factoring impacting individuals’ willingness to share information

	*Model 1: Conventional regression model*						*Model 2: Tobit regression model*					
	*Estimate*	*SE*	*P*	*Sig.*	*2.5% CI*	*97.5% CI*	*Estimate*	*SE*	*P*	*Sig.*	*2.5% CI*	*97.5% CI*
***Key independent variables***												
Intercept	-0.034	0.429	0.937		-0.875	0.808	-0.047	0.431	0.914		-0.892	0.799
Perceived helpfulness	0.127	0.019	0.000	***	0.090	0.165	0.127	0.019	0.000	***	0.089	0.165
Perceived risk	0.090	0.022	0.000	***	0.048	0.133	0.090	0.022	0.000	***	0.048	0.133
Subjective norm	0.012	0.004	0.004	**	0.004	0.020	0.012	0.004	0.004	**	0.004	0.020
Fatigue	0.034	0.015	0.026	*	0.004	0.064	0.034	0.015	0.026	*	0.004	0.064
Self-efficacy	0.011	0.006	0.051	.	0.000	0.023	0.012	0.006	0.049	*	0.00004	0.023
Promotion focus	0.144	0.019	0.000	***	0.106	0.182	0.146	0.019	0.000	***	0.108	0.183
Prevention focus	-0.018	0.024	0.462		-0.064	0.029	-0.020	0.024	0.407		-0.067	0.027
***Moderators***												
Perceived helpfulness*Promotion focus	-0.003	0.001	0.000	***	-0.005	-0.002	-0.003	0.001	0.000	***	-0.005	-0.002
Perceived risk*Promotion focus	-0.004	0.001	0.000	***	-0.006	-0.002	-0.004	0.001	0.000	***	-0.006	-0.002
Fatigue*Promotion focus	-0.001	0.001	0.169		-0.002	0.000	-0.001	0.001	0.169		-0.002	0.000
Perceived helpfulness*Prevention focus	0.001	0.001	0.409		-0.001	0.003	0.001	0.001	0.379		-0.001	0.003
Perceived risk*Prevention focus	0.002	0.001	0.070	.	0.000	0.005	0.002	0.001	0.058	.	0.000	0.005
Fatigue*Prevention focus	-0.002	0.001	0.036	*	-0.003	0.000	-0.002	0.001	0.037	*	-0.003	0.0001
***Control variables***												
**Age**												
Age (18–29)												
Age (30–39)	0.003	0.050	0.951		-0.095	0.101	0.004	0.050	0.930		-0.094	0.103
Age (40–49)	0.110	0.051	0.031	*	0.010	0.210	0.110	0.051	0.032	*	0.009	0.210
Age (50–59)	0.166	0.054	0.002	**	0.061	0.271	0.166	0.054	0.002	**	0.060	0.272
Age (≥ 60)	0.291	0.059	0.000	***	0.175	0.408	0.292	0.060	0.000	***	0.175	0.409
**Age**												
Gender (female)												
Gender (male)	-0.031	0.034	0.355		-0.097	0.035	-0.031	0.034	0.355		-0.097	0.035
**Employment**												
Employment (employed)												
Employment (unemployed)	-0.353	0.152	0.020	*	-0.650	-0.055	-0.349	0.153	0.022	*	-0.648	-0.050
**Education**												
Education (primary school and below)												
Education (high)	0.001	0.069	0.990		-0.135	0.137	-0.002	0.070	0.981		-0.138	0.135
Education (bachelor)	0.004	0.065	0.950		-0.123	0.131	0.002	0.065	0.971		-0.125	0.130
Education (master and above)	-0.127	0.110	0.248		-0.343	0.089	-0.128	0.111	0.249		-0.344	0.089
**Income**												
Income (≤ 4,999)												
Income (5,000–9,999)	-0.012	0.037	0.741		-0.086	0.061	-0.012	0.038	0.757		-0.085	0.062
Income (10,000–14,999)	0.043	0.055	0.437		-0.065	0.150	0.043	0.055	0.434		-0.065	0.151
Income (≥ 15,000)	-0.056	0.066	0.402		-0.186	0.074	-0.056	0.067	0.400		-0.187	0.075
**Digital content consumption**												
No												
Yes	-0.143	0.047	0.002	**	-0.235	-0.051	-0.146	0.047	0.002	**	-0.238	-0.054
LogSigma							0.145	0.010	< 2e-16	***	0.125	0.165

**Note**: Sig. represents the significance level: <0.001 ***, <0.01 **, <0.05 *, <0.01

We demonstrated the moderating effects of promotion and prevention focus. For each unit increase in the score for promotion focus, the impacts of perceived helpfulness and risk on the willingness to share information fell by 0.003 (95% CI: -0.01 and − 0.002) and 0.004 (95% CI: -0.01 and − 0.002), respectively. For each unit increase in the prevention focus score, the impacts of privacy fatigue on the willingness to share information fell by 0.002 (95% CI: -0.003 and − 0.0001). We also found that older individuals, the employed, and individuals who spent less than seven hours per day online were more willing to share information.

### Results of sensitivity analyses

Table 4 reports the results of subgroup analysis by the degree of economic development. We found that individuals from economically developed cities were more likely to be affected by the positive impacts of perceived risk and subjective norms on the willingness to share information. In contrast, the positive impacts of self-efficacy on the willingness to share information were more substantial among those from disadvantaged backgrounds. We still found that promotion focus reduced the positive impacts of perceived risk on the willingness to share information among individuals from well-off cities, while promotion (prevention) focus reduced the positive impacts of perceived helpfulness (privacy fatigue) on the willingness to share information among those from less privileged backgrounds.

**Table 4:** The results of subgroup analysis (stratified by cities’ economic development degree)

	*Tier 1*			*Tier 2*			*Tier 3*			*Tier 4*		
	*Estimate*	*SE*	*Sig.*	*Estimate*	*SE*	*Sig.*	*Estimate*	*SE*	*Sig.*	*Estimate*	*SE*	*Sig.*
Intercept	-0.134	0.907		-0.037	0.977		0.625	0.845		-0.213	0.814	
Perceived helpfulness	0.088	0.039	*	0.065	0.042		0.152	0.038	***	0.151	0.038	***
Perceived risk	0.107	0.044	*	0.141	0.047	**	0.033	0.042		0.071	0.043	
Subjective norm	0.037	0.009	***	0.042	0.010	***	0.004	0.009		0.008	0.007	
Fatigue	0.044	0.031		0.023	0.031		0.027	0.030		0.045	0.032	
Self-efficacy	-0.016	0.011		0.001	0.012		0.037	0.012	**	0.028	0.011	*
Promotion focus	0.134	0.044	**	0.175	0.043	***	0.121	0.037	**	0.117	0.034	***
Prevention focus	-0.069	0.048		-0.094	0.053	.	0.000	0.049		0.035	0.046	
Perceived helpfulness*Promotion focus	-0.002	0.002		-0.003	0.002	.	-0.003	0.002		-0.004	0.002	**
Perceived risk*Promotion focus	-0.005	0.002	**	-0.006	0.002	**	-0.002	0.002		-0.003	0.002	
Fatigue*Promotion focus	0.000	0.001		-0.001	0.001		-0.002	0.001		0.001	0.001	
Perceived helpfulness*Prevention focus	0.003	0.002		0.004	0.002	.	-0.002	0.002		0.001	0.002	
Perceived risk*Prevention focus	0.005	0.003	.	0.003	0.003		0.003	0.002		0.002	0.003	
Fatigue*Prevention focus	-0.003	0.002	.	0.000	0.002		-0.001	0.002		-0.005	0.002	**
**Age**												
Age (18–29)												
Age (30–39)	0.096	0.093		-0.095	0.096		-0.020	0.108		0.057	0.104	
Age (40–49)	0.074	0.096		0.177	0.106	.	0.057	0.105		0.154	0.102	
Age (50–59)	0.207	0.104	*	0.166	0.108		-0.105	0.109		0.475	0.111	***
Age (≥ 60)	0.328	0.119	**	0.060	0.119		-0.103	0.119		0.857	0.123	***
**Age**												
Gender (female)												
Gender (male)	0.021	0.066		-0.069	0.069		-0.006	0.069		-0.038	0.066	
**Employment**												
Employment (employed)												
Employment (unemployed)	-0.266	0.263		-0.412	0.258		0.270	0.417		-0.566	0.351	
**Education**												
Education (primary school and below)												
Education (high)	0.073	0.144		0.079	0.141		-0.248	0.137	.	-0.230	0.142	
Education (bachelor)	-0.129	0.171		0.161	0.257		-0.322	0.223		-0.119	0.354	
Education (master and above)	0.076	0.121		0.162	0.131		-0.201	0.129		-0.133	0.136	
**Income**												
Income (≤ 4,999)												
Income (5,000–9,999)	0.104	0.100		0.175	0.109		0.083	0.120		-0.028	0.123	
Income (10,000–14,999)	-0.040	0.114		-0.018	0.141		0.087	0.131		-0.085	0.183	
Income (≥ 15,000)	-0.043	0.078		0.125	0.075	.	0.068	0.076		-0.045	0.076	
**Digital content consumption**												
No												
Yes	-0.110	0.083		-0.231	0.095	*	-0.080	0.103		-0.137	0.096	

**Note**: Sig. represents the significance level: <0.001 ***, <0.01 **, <0.05 *, <0.01

The results of subgroup analyses by demographic characteristics were shown in Fig. B.1-B.3 in Appendix B (specific estimation results can be found in Tables B.1-B.4 in Appendix B). As shown in Fig. B.1 in Appendix B: (1) the negative impacts of self-efficacy on the willingness to share information were only found among individuals who spent more than seven hours per day online; (2) the positive impacts of privacy fatigue on the willingness to share information were most pronounced among people aged between 18 and 29 years; (3) the positive impacts of subjective norm on the willingness to share information were most noticeable among individuals who spent more than seven hours per day online; (4) the positive impacts of perceived risk on the willingness to share information were most substantial among poor individuals; and (5) individuals with a master degree and above were most likely to be affected by the positive impacts of perceived helpfulness on the willingness to share information. Across all the subgroups, prevention focus only reduced the willingness to share information among females and the less educated group (Fig. B.2, Appendix B). The less educated and young people were substantially affected by the moderating effects of prevention and promotion focus on the association between perceived risks and the willingness to share information (Fig. B.3, Appendix B). These results underline the heterogenous effects of the various factors assessed on the willingness of individuals to share information.

Table C.1 in Appendix C reports the results of the Poisson regression model. We found similar results, but a slightly smaller positive impact of perceived helpfulness, perceived risk, and promotion focus on the willingness to share information. We also found that the promotion focus reduced the positive impacts of perceived helpfulness and risk on the willingness to share information. These results suggest that our key findings were robust to different estimation strategies.

## Discussion

This study analyzed the willingness of individuals to share information with governments when adopting health technologies during the COVID-19 pandemic in China. We showed that different populations were driven by different factors to share their information with governments when using health technologies during the COVID-19 pandemic. Our results demonstrated a paradox that even when individuals perceived a high risk of sharing information and a sense of weariness toward privacy issues, they were still willing to share their information with governments when adopting health technologies during a critical health crisis.

With respect to the factors that facilitated or limited the willingness of individuals to share information with governments when adopting health technologies, we found that those who perceived a higher level of risk and privacy fatigue were more likely to share their personal information. The impacts of perceived risks and privacy fatigue on the willingness to share information somewhat reflect the privacy fatigue paradox debated in the literature [47], where even individuals who had privacy concerns about the sharing of their personal information continued to use products/services that collect their personal data. One explanation for the positive impacts of perceived risk may be biased risk perception that results in behavioral decisions that deviate from the ration norm [48]. Another reason that explains the positive impacts of perceived risk on the willingness to share information may lie in that COVID-19 perceived risks were strongly associated with prosocial values whereby people were obliged to do things for the benefit of society even when such things come with a cost at a personal level [49]. The positive impacts of privacy fatigue on the willingness to share information might be explained by the unwillingness of individuals to give up convenient behaviors, even when they are associated with the sharing of information, and the Chinese cultural tradition of collectivism. Several studies have assessed the role of collectivism in China and have concluded that Chinese citizens often placed greater priority to collective rather than personal goals and aspirations [50–52]. These findings from the literature suggests that Chinese citizens may be more compliant and subjugate their personal privacy concerns with respect to information sharing for the potential greater good for public health [53].

This study also demonstrated that promotion (prevention) focus reduced the positive impacts of perceived helpfulness/risk (privacy fatigue) on the willingness to share information with governments when adopting health technologies. Promotion focus reduced the positive impacts of perceived helpfulness and risk on the willingness to share information, which aligns with prior studies demonstrating that people with a promotion focus were motivated by rewards and achievements [49, 54]. On the other hand, we found that prevention focus reduced the association between privacy fatigue and the willingness to share information, which is not surprising since people with a prevention focus generally favor the status quo while avoiding unnecessary risks [49].

Our subgroup analyses offered interesting results. We demonstrated that the positive association between privacy fatigue and the willingness to share information was most substantial among young adults aged between 18 and 29 years, which is in line with previous studies [17, 55, 56]. We also found that prevention focus only reduced the willingness to share information for women when compared with their counterparts. This evidence relates to previous COVID-19 studies that convincingly indicate strong differences between males’ and females’ beliefs during the pandemic [57]. These prior studies demonstrated that females may be more risk-aversive [58, 59], and hence, placed significant importance on the negative consequences of sharing information [60, 61]. Across all subgroups, self-efficacy was only found to reduce the willingness to share information among individuals who spent more than seven hours per day online. It has been evidently demonstrated that advancement in digital technology contributes to a dramatic rise in online media consumption [62] and time spent online has increased drastically during the COVID-19 pandemic [30]. Excessive digital content consumption may give rise to an increased level of sadness, anxiety, negative emotions, and uncertainty [56, 63], which may in turn reduce the positive impacts of self-efficacy on the willingness to share information.

The results of this study provide policy insights into facilitating digital governance for governments as they plan to motivate their populace to share their information during a health crisis. As governments plan to leverage the use of specific digital governance interventions during pandemics, this study has demonstrated that there are benefits in the development of strategies that protect sensitive personal information. Such strategies may build public confidence in accountability and reduce the chance of possible data breaches. More importantly, given the dire need for digital governance during pandemics, a carrot-and-stick approach may be employed by policymakers to encourage citizens to share necessary personal information. For example, a “stick approach” could be used for those involved in data breaches, while a “carrot approach” used to attract particular population sub-groups, such as the elderly, to provide customized and tailored information.

This study has some strengths compared with other investigations carried out in this field. Although several studies have explored the determinants of information-sharing intentions, there remains a paucity of research exploring the motivations of individuals to share information with their government when adopting health technologies during the COVID-19 pandemic in China. To the best of our knowledge, this is the first study conducted in China to comprehensively assess the impacts of perceived helpfulness, perceived risk, self-efficacy, subjective norm, privacy fatigue, promotion focus, and prevention focus on the willingness of individuals to share information with their government when using health technologies during the COVID-19 pandemic. Our research findings contribute to the extant literature by adding evidence on the unexpected association between perceived risk and willingness to share information. Another valuable contribution of our study is that when confronting a critical health crisis, individuals were motivated by different reasons to share their information with the government. This study also has some practical implications in terms of offering guidance for policy decision-makers who wish to collect personal information from citizens to manage a health crisis.

Several limitations warrant consideration when interpreting our study findings. First,  a cross-sectional design was used to collect the study data which limits our ability to directly address issues of causality. Nevertheless, cross-sectional designs offer a basis for potential correlations among variables from which future causal relationships may be drawn. Second, our sample of respondents was drawn from relatively economically developed cities from Eastern China, which may threaten the generalizability of the study findings to other, low-income settings. However, this study did not discover systematical differences in the willingness to share information across populations with different income levels. We therefore expected that our study findings may be generalized to both low- and high-income settings, although more rigorous conclusions cannot be reached before examining these associations using samples of different populations. Third, since our study opted for face-to-face interviews, another limitation of this study was the potential social desirability bias that the sampled individuals may underreport socially undesirable activities and overreport socially desirable ones [64]. Nevertheless, such bias would have been greater if we did not make the questionnaire anonymous and use non-leading questions that are free from any potential influence. Finally, limited by data availability, we were unable to analyze the pathways that explain the complex association between and among the proposed determinants. As such, we strongly recommend future studies with detailed information be used to explore the underlying mechanism through which perceived helpfulness, perceived risk, subjective norm, and privacy fatigue impact the willingness of individuals to share information with governments when adopting health technologies.

## Conclusions

This study demonstrated that perceived helpfulness, perceived risk, subjective norm, and privacy fatigue increased the willingness of individuals to share information with governments when using health technologies during the COVID-19 pandemic in China. This work highlights the presence of heterogeneity in the impacts of the proposed factors on the willingness to share information during a pandemic. Policy decision-makers are therefore suggested to propose targeted policies when confronting challenges in collecting information from individuals in order to combat a critical health crisis.

### Electronic supplementary material

Below is the link to the electronic supplementary material.

Supplementary Material 1

## Data Availability

The datasets generated and/or analysed during the current study are not publicly available due to privacy concerns but are available from the corresponding author on reasonable request.

## Abbreviations

TRA: Theory of reasoned action
TPB: Theory of Planned Behavior
PCT: Privacy calculus theory
RFT: Regulatory focus theory

## Footnotes

1 All the questionnaires were completed within one month to make sure that the levels of perceived risk and helpfulness would not change dynamically in terms of how the government reacted to the COVID-19 pandemic.

2 This indicates that our study samples are valid as a result of administering the questionnaires in person and professional training towards all the researchers.
