# Supplementary material for: What motivates individuals to share information with governments when adopting health technologies during the COVID-19 pandemic?
Source: BMC Public Health. 2023 Dec 18;23:2527. doi: 10.1186/s12889-023-17437-2 (PMC10726615; doi:10.1186/s12889-023-17437-2)
Supplement: Supplementary file 1 — Supplementary Material 1 [file 12889_2023_17437_MOESM1_ESM.docx]

# Appendix

**Appendix A**

**Table A.1 Measurement scales**

| **Variables** | **Items** | **Score range** |
| --- | --- | --- |
| **Dependent variable** | |  |
| WTS | 1. Are you willing to share information with the governments when using health technologies during the pandemic? | 0-7 |
| **Independent variables** | |  |
| Perceived helpfulness | 1. Providing personal information is helpful 2. Providing personal information helps me enjoy the service I want 3. Providing personal information offers me timely and accurate information 4. Providing personal information enables me to better interact and connect with individuals who share the same interests and inspirations | 0-28 |
| Perceived risk | 1. Personal information provided is highly likely to be breached 2. Personal information provided may be inappropriately collected and misused 3. Providing personal information may lead to unpredictable consequences | 0-21 |
| Subjective norm | 1. Most of my friends attach great importance to the protection of personal information 2. Most of my friends are cautious about reporting their personal information 3. Most of my friends think that I should pay attention to the protection of personal information 4. Most of my friends think that I should be careful about reporting my personal information | 0-28 |
| Fatigue | 1. I am tired of dealing with issues related to the protection of personal information 2. I do not care about issues related to the protection of personal information 3. I am less interested in issues related to the protection of personal information 4. I am less passionate about offering personal information | 0-28 |
| Self-efficacy | 1. I have competence in protecting my personal information 2. I have skills to prevent personal information breaches 3. I know how to address potential threats from information breaches | 0-21 |
| **Moderators** | | |
| Promotion focus | 1. Compared to most people, are you typically unable to get what you want out of life? 2. I feel like I have made progress toward being successful in my life 3. When it comes to achieving things that are important to me, I find that I don’t perform as well as I ideally would like to do 4. Do you often do well at things that you try? 5. I found every few hobbies or activities in my life that capture my interest or motivate me to put efforts into them 6. How often have you accomplished things that got you “psyched” to work even harder? | 0-42 |
| Prevention focus | 1. Growing up, would you ever “cross the line” by doing things that your parents would not tolerate? 2. Did you get on your parents’ nerves often when you were growing up? 3. How often did you obey rules and regulations that were established by your parents? 4. Growing up, did you ever act in ways that your parents’ thought were objectionable? | 0-28 |

**Note:** All the items included in each measurement scale were measured by a Seven-point Likert scale.

**Appendix B – Results of subgroup analyses**

**Fig. B.1 The results of subgroup analyses (self-efficacy, fatigue, subjective norm, perceived risk, and perceived helpfulness)**

**Fig. B.2 The results of subgroup analyses (promotion and prevention focus)**

**Fig. B.3 The results of subgroup analyses (moderators)**

**Table B.1 Results of subgroup analysis by age**

|  | **Age: ≤29** | | | **Age: 30-39** | | | **Age: 40-49** | | | **Age: 50-59** | | | **Age: ≥60** | | |
| --- | --- | --- | --- | --- | --- | --- | --- | --- | --- | --- | --- | --- | --- | --- | --- |
|  | **Estimate** | **SE** | **Sig.** | **Estimate** | **SE** | **Sig.** | **Estimate** | **SE** | **Sig.** | **Estimate** | **SE** | **Sig.** | **Estimate** | **SE** | **Sig.** |
| Intercept | -0.208 | 1.019 |  | -1.907 | 0.886 | * | 0.710 | 0.935 |  | 0.364 | 1.054 |  | 0.358 | 1.062 |  |
| Perceived helpfulness | 0.100 | 0.044 | * | 0.191 | 0.039 | *** | 0.060 | 0.039 |  | 0.200 | 0.049 | *** | 0.160 | 0.053 | ** |
| Perceived risk | 0.102 | 0.050 | * | 0.174 | 0.044 | *** | 0.120 | 0.047 | * | -0.025 | 0.053 |  | 0.071 | 0.054 |  |
| Subjective norm | 0.027 | 0.011 | * | 0.021 | 0.009 | * | 0.013 | 0.009 |  | 0.024 | 0.009 | ** | -0.014 | 0.010 |  |
| Fatigue | 0.069 | 0.034 | * | 0.001 | 0.031 |  | 0.015 | 0.033 |  | 0.053 | 0.037 |  | 0.031 | 0.041 |  |
| Self-efficacy | 0.001 | 0.013 |  | -0.010 | 0.013 |  | 0.032 | 0.012 | ** | -0.002 | 0.014 |  | 0.024 | 0.017 |  |
| Promotion focus | 0.192 | 0.050 | *** | 0.206 | 0.041 | *** | 0.147 | 0.041 | *** | 0.157 | 0.046 | *** | 0.045 | 0.045 |  |
| Prevention focus | -0.094 | 0.059 |  | 0.000 | 0.050 |  | -0.060 | 0.052 |  | -0.052 | 0.053 |  | 0.136 | 0.062 | * |
| Perceived helpfulness*Promotion focus | -0.004 | 0.002 | . | -0.005 | 0.002 | ** | -0.003 | 0.002 |  | -0.006 | 0.002 | ** | 0.000 | 0.002 |  |
| Perceived risk*Promotion focus | -0.008 | 0.002 | ** | -0.007 | 0.002 | *** | -0.006 | 0.002 | *** | 0.000 | 0.002 |  | 0.000 | 0.002 |  |
| Fatigue*Promotion focus | -0.001 | 0.002 |  | 0.000 | 0.001 |  | 0.002 | 0.001 |  | -0.003 | 0.002 | * | -0.002 | 0.002 |  |
| Perceived helpfulness*Prevention focus | 0.003 | 0.002 |  | 0.000 | 0.002 |  | 0.004 | 0.002 | . | 0.001 | 0.002 |  | -0.006 | 0.003 | . |
| Perceived risk*Prevention focus | 0.007 | 0.003 | * | 0.002 | 0.003 |  | 0.004 | 0.003 |  | 0.003 | 0.003 |  | -0.003 | 0.003 |  |
| Fatigue*Prevention focus | -0.005 | 0.002 | ** | 0.000 | 0.002 |  | -0.005 | 0.002 | * | 0.001 | 0.002 |  | 0.000 | 0.002 |  |
| Employment (employed) |  |  |  |  |  |  |  |  |  |  |  |  |  |  |  |
| Employment (unemployed) | -0.663 | 0.264 | * | 0.419 | 0.406 |  | -0.017 | 0.275 |  | -0.243 | 0.417 |  | -0.881 | 0.566 |  |
| Online (no) |  |  |  |  |  |  |  |  |  |  |  |  |  |  |  |
| Online (yes) | 0.008 | 0.100 |  | -0.218 | 0.089 | * | -0.112 | 0.097 |  | -0.364 | 0.114 | ** | 0.006 | 0.145 |  |
| Gender (female) |  |  |  |  |  |  |  |  |  |  |  |  |  |  |  |
| Gender (male) | -0.094 | 0.074 |  | 0.009 | 0.065 |  | -0.012 | 0.070 |  | -0.057 | 0.082 |  | 0.020 | 0.094 |  |
| Education (primary school and below) |  |  |  |  |  |  |  |  |  |  |  |  |  |  |  |
| Education (high) | -0.012 | 0.157 |  | 0.033 | 0.150 |  | -0.285 | 0.154 | . | 0.043 | 0.158 |  | 0.186 | 0.185 |  |
| Education (bachelor) | 0.052 | 0.126 |  | 0.115 | 0.139 |  | -0.138 | 0.143 |  | -0.001 | 0.154 |  | 0.017 | 0.185 |  |
| Education (master and above) | -0.134 | 0.192 |  | 0.099 | 0.197 |  | -0.534 | 0.260 | * | -0.204 | 0.431 |  | 0.333 | 0.427 |  |
| Income (≤4,999) |  |  |  |  |  |  |  |  |  |  |  |  |  |  |  |
| Income (5,000-9,999) | -0.018 | 0.081 |  | -0.011 | 0.073 |  | -0.004 | 0.076 |  | -0.067 | 0.093 |  | 0.049 | 0.108 |  |
| Income (10,000-14,999) | 0.182 | 0.130 |  | -0.121 | 0.101 |  | 0.190 | 0.120 |  | 0.087 | 0.133 |  | -0.135 | 0.146 |  |
| Income (≥15,000) | 0.082 | 0.164 |  | -0.127 | 0.120 |  | -0.174 | 0.143 |  | 0.070 | 0.158 |  | -0.103 | 0.179 |  |

**Note:** Significance. codes: ＜0.001 ***, ＜0.01 **, ＜0.05 *, ＜0.01.

**Table B.2 The results of subgroup analysis by education**

|  | **Primary and below** | | | **High** | | | **Bachelor** | | | **Master and above** | | |
| --- | --- | --- | --- | --- | --- | --- | --- | --- | --- | --- | --- | --- |
|  | **Estimate** | **SE** | **Sig.** | **Estimate** | **SE** | **Sig.** | **Estimate** | **SE** | **Sig.** | **Estimate** | **SE** | **Sig.** |
| Intercept | 1.865 | 1.507 |  | -0.822 | 0.844 |  | 0.236 | 0.548 |  | -1.585 | 2.314 |  |
| Perceived helpfulness | 0.013 | 0.069 |  | 0.213 | 0.038 | *** | 0.090 | 0.025 | *** | 0.279 | 0.101 | ** |
| Perceived risk | 0.057 | 0.072 |  | 0.061 | 0.044 |  | 0.109 | 0.028 | *** | 0.150 | 0.106 |  |
| Subjective norm | 0.006 | 0.014 |  | 0.009 | 0.008 |  | 0.016 | 0.005 | ** | 0.005 | 0.025 |  |
| Fatigue | 0.038 | 0.052 |  | 0.016 | 0.031 |  | 0.046 | 0.019 | * | -0.099 | 0.080 |  |
| Self-efficacy | 0.037 | 0.022 | . | 0.021 | 0.012 | . | 0.005 | 0.007 |  | 0.002 | 0.037 |  |
| Promotion focus | 0.169 | 0.074 | * | 0.117 | 0.036 | ** | 0.155 | 0.025 | *** | 0.136 | 0.100 |  |
| Prevention focus | -0.168 | 0.081 | * | 0.088 | 0.047 | . | -0.059 | 0.031 | . | 0.067 | 0.129 |  |
| Perceived helpfulness*Promotion focus | 0.001 | 0.003 |  | -0.005 | 0.002 | ** | -0.002 | 0.001 | * | -0.006 | 0.004 |  |
| Perceived risk*Promotion focus | -0.010 | 0.003 | ** | 0.000 | 0.002 |  | -0.005 | 0.001 | *** | -0.008 | 0.005 | . |
| Fatigue*Promotion focus | -0.001 | 0.003 |  | -0.001 | 0.001 |  | -0.001 | 0.001 |  | 0.007 | 0.003 | * |
| Perceived helpfulness*Prevention focus | 0.000 | 0.003 |  | -0.002 | 0.002 |  | 0.002 | 0.001 | . | -0.002 | 0.005 |  |
| Perceived risk*Prevention focus | 0.015 | 0.005 | ** | -0.003 | 0.003 |  | 0.003 | 0.002 | * | 0.003 | 0.007 |  |
| Fatigue*Prevention focus | -0.002 | 0.003 |  | -0.002 | 0.002 |  | -0.002 | 0.001 |  | -0.006 | 0.005 |  |
| Employment (employed) |  |  |  |  |  |  |  |  |  |  |  |  |
| Employment (unemployed) | -1.215 | 0.562 | * | -0.469 | 0.298 |  | -0.197 | 0.191 |  | 0.822 | 0.996 |  |
| Online (no) |  |  |  |  |  |  |  |  |  |  |  |  |
| Online (yes) | -0.191 | 0.168 |  | -0.064 | 0.093 |  | -0.202 | 0.059 | *** | 0.289 | 0.237 |  |
| Gender (female) |  |  |  |  |  |  |  |  |  |  |  |  |
| Gender (male) | 0.008 | 0.119 |  | -0.064 | 0.067 |  | -0.024 | 0.042 |  | 0.038 | 0.195 |  |
| Age (18-29) |  |  |  |  |  |  |  |  |  |  |  |  |
| Age (30-39) | -0.036 | 0.181 |  | -0.040 | 0.133 |  | 0.002 | 0.059 |  | 0.199 | 0.228 |  |
| Age (40-49) | 0.301 | 0.175 | . | 0.020 | 0.129 |  | 0.127 | 0.060 | * | 0.038 | 0.294 |  |
| Age (50-59) | 0.176 | 0.172 |  | 0.195 | 0.124 |  | 0.136 | 0.067 | * | 0.156 | 0.430 |  |
| Age (≥60) | 0.199 | 0.193 |  | 0.431 | 0.128 | *** | 0.160 | 0.079 | * | 0.752 | 0.453 | . |
| Income (≤4,999) |  |  |  |  |  |  |  |  |  |  |  |  |
| Income (5,000-9,999) | -0.087 | 0.135 |  | -0.030 | 0.076 |  | 0.004 | 0.046 |  | -0.350 | 0.234 |  |
| Income (10,000-14,999) | 0.048 | 0.193 |  | -0.092 | 0.122 |  | 0.096 | 0.066 |  | -0.422 | 0.340 |  |
| Income (≥15,000) | -0.371 | 0.215 | . | -0.109 | 0.127 |  | 0.029 | 0.089 |  | -0.498 | 0.280 | . |

**Note:** Significance. codes: ＜0.001 ***, ＜0.01 **, ＜0.05 *, ＜0.01.

**Table B.3 The results of subgroup analysis (income)**

|  | **≤4,999 CNY** | | | **5,000-9,999 CNY** | | | **10,000-14,999 CNY** | | | **≥15,000 CNY** | | |
| --- | --- | --- | --- | --- | --- | --- | --- | --- | --- | --- | --- | --- |
|  | **Estimate** | **SE** | **Sig.** | **Estimate** | **SE** | **Sig.** | **Estimate** | **SE** | **Sig.** | **Estimate** | **SE** | **Sig.** |
| Intercept | 1.758 | 1.276 |  | 1.868 | 1.813 |  | 0.426 | 0.623 |  | -1.728 | 0.756 | * |
| Perceived helpfulness | -0.010 | 0.062 |  | 0.105 | 0.084 |  | 0.106 | 0.028 | *** | 0.201 | 0.033 | *** |
| Perceived risk | 0.168 | 0.067 | * | -0.115 | 0.085 |  | 0.076 | 0.032 | * | 0.145 | 0.036 | *** |
| Subjective norm | 0.015 | 0.012 |  | -0.005 | 0.017 |  | 0.017 | 0.006 | ** | 0.007 | 0.007 |  |
| Fatigue | 0.010 | 0.046 |  | 0.069 | 0.058 |  | 0.036 | 0.023 |  | 0.034 | 0.026 |  |
| Self-efficacy | 0.008 | 0.019 |  | 0.012 | 0.020 |  | 0.016 | 0.009 | . | 0.002 | 0.010 |  |
| Promotion focus | 0.074 | 0.059 |  | 0.132 | 0.076 | . | 0.141 | 0.028 | *** | 0.177 | 0.034 | *** |
| Prevention focus | -0.040 | 0.073 |  | -0.088 | 0.089 |  | -0.045 | 0.036 |  | 0.048 | 0.041 |  |
| Perceived helpfulness*Promotion focus | 0.001 | 0.002 |  | -0.001 | 0.003 |  | -0.004 | 0.001 | ** | -0.004 | 0.001 | ** |
| Perceived risk*Promotion focus | -0.003 | 0.003 |  | 0.001 | 0.003 |  | -0.005 | 0.001 | *** | -0.004 | 0.002 | * |
| Fatigue*Promotion focus | -0.001 | 0.002 |  | -0.004 | 0.003 |  | 0.001 | 0.001 |  | -0.002 | 0.001 | * |
| Perceived helpfulness*Prevention focus | 0.005 | 0.003 |  | -0.003 | 0.004 |  | 0.003 | 0.002 | . | -0.001 | 0.002 |  |
| Perceived risk*Prevention focus | -0.004 | 0.004 |  | 0.008 | 0.005 | . | 0.005 | 0.002 | * | -0.002 | 0.002 |  |
| Fatigue*Prevention focus | 0.000 | 0.003 |  | 0.003 | 0.003 |  | -0.005 | 0.001 | *** | 0.001 | 0.001 |  |
| Employment (employed) |  |  |  |  |  |  |  |  |  |  |  |  |
| Employment (unemployed) | -2.851 | 0.850 | *** | 0.585 | 0.840 |  | -0.358 | 0.192 | . | -0.143 | 0.288 |  |
| Online (no) |  |  |  |  |  |  |  |  |  |  |  |  |
| Online (yes) | -0.197 | 0.136 |  | -0.055 | 0.156 |  | -0.196 | 0.073 | ** | -0.080 | 0.077 |  |
| Gender (female) |  |  |  |  |  |  |  |  |  |  |  |  |
| Gender (male) | 0.006 | 0.097 |  | -0.047 | 0.129 |  | -0.024 | 0.052 |  | -0.053 | 0.054 |  |
| Education (primary school and below) |  |  |  |  |  |  |  |  |  |  |  |  |
| Education (high) | -0.071 | 0.206 |  | 0.281 | 0.237 |  | -0.034 | 0.107 |  | -0.025 | 0.116 |  |
| Education (bachelor) | -0.023 | 0.187 |  | 0.407 | 0.223 | . | -0.053 | 0.101 |  | -0.020 | 0.106 |  |
| Education (master and above) | -0.440 | 0.319 |  | 0.066 | 0.308 |  | 0.013 | 0.174 |  | -0.224 | 0.192 |  |
| Age (18-29) |  |  |  |  |  |  |  |  |  |  |  |  |
| Age (30-39) | -0.296 | 0.148 | * | -0.136 | 0.193 |  | 0.070 | 0.078 |  | 0.054 | 0.080 |  |
| Age (40-49) | 0.122 | 0.159 |  | -0.191 | 0.206 |  | 0.135 | 0.077 | . | 0.132 | 0.081 |  |
| Age (50-59) | 0.077 | 0.162 |  | 0.097 | 0.210 |  | 0.216 | 0.081 | ** | 0.132 | 0.088 |  |
| Age (≥60) | -0.021 | 0.172 |  | 0.042 | 0.222 |  | 0.333 | 0.090 | *** | 0.396 | 0.100 | *** |

**Note:** Significance. codes: ＜0.001 ***, ＜0.01 **, ＜0.05 *, ＜0.01.

**Table B.4 The results of subgroup analyses (online, employment, and gender)**

|  | **Online: Yes** | | | **Online: No** | | | **Employed** | | | **Unemployed** | | | **Female** | | | **Male** | | |
| --- | --- | --- | --- | --- | --- | --- | --- | --- | --- | --- | --- | --- | --- | --- | --- | --- | --- | --- |
|  | **Estimate** | **SE** | **Sig.** | **Estimate** | **SE** | **Sig.** | **Estimate** | **SE** | **Sig.** | **Estimate** | **SE** | **Sig.** | **Estimate** | **SE** | **Sig.** | **Estimate** | **SE** | **Sig.** |
| Intercept | -0.108 | 1.1 |  | -0.117 | 0.47 |  | -0.145 | 0.434 |  | 2.678 | 5.557 |  | 1.1 | 0.582 | . | -1.147 | 0.641 | . |
| Perceived helpfulness | 0.134 | 0.047 | ** | 0.133 | 0.021 | *** | 0.134 | 0.02 | *** | 0.071 | 0.309 |  | 0.077 | 0.027 | ** | 0.17 | 0.028 | *** |
| Perceived risk | 0.068 | 0.056 |  | 0.094 | 0.023 | *** | 0.095 | 0.022 | *** | -0.239 | 0.378 |  | 0.102 | 0.029 | *** | 0.091 | 0.032 | ** |
| Subjective norm | 0.039 | 0.012 | ** | 0.008 | 0.004 | . | 0.012 | 0.004 | ** | 0.03 | 0.057 |  | 0.009 | 0.006 |  | 0.013 | 0.006 | * |
| Fatigue | 0.033 | 0.038 |  | 0.034 | 0.017 | * | 0.032 | 0.016 | * | 0.113 | 0.192 |  | 0.02 | 0.02 |  | 0.052 | 0.023 | * |
| Self-efficacy | -0.049 | 0.016 | ** | 0.021 | 0.006 | *** | 0.011 | 0.006 | . | -0.022 | 0.079 |  | 0.004 | 0.008 |  | 0.017 | 0.008 | * |
| Promotion focus | 0.058 | 0.05 |  | 0.161 | 0.021 | *** | 0.153 | 0.02 | *** | -0.044 | 0.149 |  | 0.138 | 0.026 | *** | 0.152 | 0.029 | *** |
| Prevention focus | 0.078 | 0.06 |  | -0.032 | 0.026 |  | -0.02 | 0.024 |  | -0.165 | 0.343 |  | -0.094 | 0.033 | ** | 0.058 | 0.035 |  |
| Perceived helpfulness*Promotion focus | 0.000 | 0.002 |  | -0.004 | 0.001 | *** | -0.003 | 0.001 | *** | -0.015 | 0.012 |  | -0.003 | 0.001 | * | -0.004 | 0.001 | ** |
| Perceived risk*Promotion focus | -0.001 | 0.002 |  | -0.005 | 0.001 | *** | -0.004 | 0.001 | *** | 0.02 | 0.014 |  | -0.005 | 0.001 | *** | -0.003 | 0.001 | * |
| Fatigue*Promotion focus | 0.000 | 0.002 |  | -0.001 | 0.001 |  | -0.001 | 0.001 |  | 0.004 | 0.007 |  | 0 | 0.001 |  | -0.002 | 0.001 | . |
| Perceived helpfulness*Prevention focus | -0.002 | 0.002 |  | 0.001 | 0.001 |  | 0.001 | 0.001 |  | 0.021 | 0.013 |  | 0.004 | 0.001 | ** | -0.002 | 0.001 |  |
| Perceived risk*Prevention focus | -0.001 | 0.003 |  | 0.003 | 0.001 | * | 0.003 | 0.001 | * | -0.002 | 0.024 |  | 0.003 | 0.002 | . | 0.001 | 0.002 |  |
| Fatigue*Prevention focus | -0.001 | 0.002 |  | -0.002 | 0.001 | * | -0.002 | 0.001 | . | -0.015 | 0.015 |  | -0.002 | 0.001 |  | -0.002 | 0.001 |  |
| Employment (employed) |  |  |  |  |  |  |  |  |  |  |  |  |  |  |  |  |  |  |
| Employment (unemployed) | -0.013 | 0.36 |  | -0.433 | 0.169 | * |  |  |  |  |  |  | -0.557 | 0.212 | ** | -0.191 | 0.218 |  |
| Online (no) |  |  |  |  |  |  |  |  |  |  |  |  |  |  |  |  |  |  |
| Online (yes) |  |  |  |  |  |  | -0.147 | 0.047 | ** | 0.511 | 0.63 |  | -0.128 | 0.064 | * | -0.166 | 0.069 | * |
| Gender (female) |  |  |  |  |  |  |  |  |  |  |  |  |  |  |  |  |  |  |
| Gender (male) | -0.044 | 0.096 |  | -0.023 | 0.036 |  | -0.034 | 0.034 |  | -0.429 | 0.514 |  |  |  |  |  |  |  |
| Education (primary school and below) |  |  |  |  |  |  |  |  |  |  |  |  |  |  |  |  |  |  |
| Education (high) | 0.09 | 0.197 |  | -0.031 | 0.074 |  | -0.008 | 0.07 |  | 0.499 | 0.974 |  | 0.063 | 0.093 |  | -0.074 | 0.104 |  |
| Education (bachelor) | -0.02 | 0.183 |  | -0.001 | 0.069 |  | -0.008 | 0.065 |  | 1.629 | 1.003 |  | 0.015 | 0.086 |  | -0.025 | 0.098 |  |
| Education (master and above) | 0.188 | 0.273 |  | -0.21 | 0.121 | . | -0.138 | 0.11 |  | -0.277 | 1.863 |  | -0.212 | 0.157 |  | -0.097 | 0.157 |  |
| Age (18-29) |  |  |  |  |  |  |  |  |  |  |  |  |  |  |  |  |  |  |
| Age (30~39) | -0.165 | 0.137 |  | 0.043 | 0.054 |  | -0.007 | 0.05 |  | 1.107 | 0.739 |  | -0.05 | 0.066 |  | 0.062 | 0.077 |  |
| Age (40~49) | 0.023 | 0.142 |  | 0.138 | 0.054 | * | 0.099 | 0.051 | . | 1.726 | 0.632 | * | 0.059 | 0.071 |  | 0.175 | 0.073 | * |
| Age (50~59) | -0.125 | 0.151 |  | 0.222 | 0.057 | *** | 0.157 | 0.054 | ** | 0.828 | 0.821 |  | 0.138 | 0.074 | . | 0.207 | 0.079 | ** |
| Age (≥60) | 0.278 | 0.176 |  | 0.306 | 0.063 | *** | 0.291 | 0.059 | *** | 0.37 | 1.013 |  | 0.207 | 0.081 | * | 0.392 | 0.087 | *** |
| Income (≤4,999) |  |  |  |  |  |  |  |  |  |  |  |  |  |  |  |  |  |  |
| Income (5,000-9,999) | 0.13 | 0.108 |  | -0.03 | 0.04 |  | -0.014 | 0.037 |  | 0.254 | 0.535 |  | -0.001 | 0.052 |  | -0.032 | 0.054 |  |
| Income (10,000-14,999) | 0.124 | 0.157 |  | 0.033 | 0.058 |  | 0.05 | 0.055 |  | -2.66 | 1.501 | . | 0.024 | 0.076 |  | 0.05 | 0.08 |  |
| Income (≥15,000) | 0.009 | 0.162 |  | -0.055 | 0.073 |  | -0.058 | 0.066 |  | -0.771 | 1.41 |  | -0.03 | 0.091 |  | -0.079 | 0.098 |  |

**Note:** Significance. codes: ＜0.001 ***, ＜0.01 **, ＜0.05 *, ＜0.01.

**Appendix C**

**Table C.1 The results of Poisson regression model**

|  | **Estimate** | **Std. Error** | **Pr(>\|t\|)** | **Sig.** | **2.5% CI** | **97.5% CI** |
| --- | --- | --- | --- | --- | --- | --- |
| (Intercept) | 0.444 | 0.175 | 0.011 | * | 0.101 | 0.787 |
| Perceived helpfulness | 0.032 | 0.008 | 0.000 | *** | 0.017 | 0.048 |
| Perceived risk | 0.021 | 0.009 | 0.017 | * | 0.004 | 0.038 |
| Subjective norm | 0.003 | 0.002 | 0.119 |  | -0.001 | 0.006 |
| Fatigue | 0.006 | 0.006 | 0.351 |  | -0.006 | 0.018 |
| Self-efficacy | 0.002 | 0.002 | 0.315 |  | -0.002 | 0.007 |
| Promotion focus | 0.035 | 0.008 | 0.000 | *** | 0.02 | 0.051 |
| Prevention focus | -0.006 | 0.01 | 0.564 |  | -0.025 | 0.014 |
| Perceived helpfulness*Promotion focus | -0.001 | 0.000 | 0.008 | ** | -0.002 | 0.000 |
| Perceived risk*Promotion focus | -0.001 | 0.000 | 0.009 | ** | -0.002 | 0.000 |
| Fatigue*Promotion focus | 0.000 | 0.000 | 0.616 |  | -0.001 | 0.000 |
| Perceived helpfulness*Prevention focus | 0.000 | 0.000 | 0.586 |  | -0.001 | 0.001 |
| Perceived risk*Prevention focus | 0.001 | 0.001 | 0.276 |  | 0.000 | 0.002 |
| Fatigue*Prevention focus | 0.000 | 0.000 | 0.29 |  | -0.001 | 0.000 |
| Age (18-29) |  |  |  |  |  |  |
| Age (30-39) | 0.001 | 0.02 | 0.976 |  | -0.039 | 0.040 |
| Age (40~49) | 0.024 | 0.021 | 0.253 |  | -0.017 | 0.064 |
| Age (50~59) | 0.036 | 0.022 | 0.101 |  | -0.007 | 0.078 |
| Age (≥60) | 0.063 | 0.024 | 0.009 | ** | 0.016 | 0.109 |
| Gender (female) |  |  |  |  |  |  |
| Gender (male) | -0.006 | 0.014 | 0.635 |  | -0.033 | 0.02 |
| Employment (employed) |  |  |  |  |  |  |
| Employment (unemployed) | -0.083 | 0.065 | 0.202 |  | -0.214 | 0.042 |
| Education (primary school and below) |  |  |  |  |  |  |
| Education (high) | 0.001 | 0.028 | 0.979 |  | -0.054 | 0.056 |
| Education (master and above) | -0.029 | 0.045 | 0.52 |  | -0.117 | 0.058 |
| Education (bachelor) | 0.001 | 0.026 | 0.966 |  | -0.05 | 0.053 |
| Income (≤4,999) |  |  |  |  |  |  |
| Income (5,000-10,000) | -0.002 | 0.015 | 0.883 |  | -0.032 | 0.027 |
| Income (10,000-14,999) | 0.009 | 0.022 | 0.68 |  | -0.034 | 0.052 |
| Income (≥15,000) | -0.012 | 0.026 | 0.646 |  | -0.064 | 0.039 |
| Online (no) |  |  |  |  |  |  |
| Online (yes) | -0.031 | 0.019 | 0.100 | . | -0.069 | 0.006 |

**Note:** Significance. codes: ＜0.001 ***, ＜0.01 **, ＜0.05 *, ＜0.01.
